# Prevalence of Idiopathic Macular Hole in a Specialized Ophthalmological Referral Center: A Retrospective Cross-Sectional Study (2018-2022)

**DOI:** 10.7759/cureus.75821

**Published:** 2024-12-16

**Authors:** Gerardo Ledesma-Gil, Braulio Hernan Velasco Sepulveda, Paulina Bueno Zarazua, Armando Bautista Barba, David Vega-Morales, Federico Graue Wiechers, Denise Loya Garcia, Martin A Mainster

**Affiliations:** 1 Retina Department, Institute of Ophthalmology Fundacion Conde de Valenciana, Mexico City, MEX; 2 School of Medicine and Health Sciences, Tecnológico de Monterrey, Monterrey, MEX; 3 Ophthalmology Department, Institute of Ophthalmology Fundacion Conde de Valenciana, Mexico City, MEX; 4 Rheumatology Department, Instituto Mexicano del Seguro Social, Monterrey, MEX; 5 Ophthalmology Department, University of Kansas School of Medicine, Kansas City, USA

**Keywords:** hispanic population, idiopathic macular hole, optical coherence tomography (oct), prevalence, watzke-allen sign

## Abstract

Background: An idiopathic macular hole (IMH) is a foveal opening in the neurosensory retina caused by perifoveal vitreomacular traction and detachment. IMH prevalence varies considerably across populations, highlighting a need for further investigation, especially in underrepresented groups such as Hispanics.

Methods: This retrospective, descriptive, cross-sectional study analyzed IMH prevalence in a Hispanic population over four years. Electronic records of patients were reviewed at a single referral center. All patients aged 18 years and above who presented for a first-time comprehensive ophthalmology evaluation were included. IMH diagnosis was clinically made and confirmed with optical coherence tomography (OCT). Data analysis involved descriptive and inferential statistics, adhering to Strengthening the Reporting of Observational Studies in Epidemiology (STROBE) guidelines.

Results: Of 116,655 electronic records analyzed from March 2018 to July 2022, 397 subjects were identified in our analysis. The estimated prevalence of IMH was 0.34% (95% confidence interval (CI): 0.307-0.375), with a female-to-male ratio of 4:1. The mean age of the participants was 67.75 ± 9.38 years. Initial visual acuity, laterality, comorbidities, and clinical findings were assessed. No significant gender differences were documented. Subgroup analyses revealed significant age discrepancies based on comorbidities and clinical symptoms.

Conclusions: Our study provides insight into the prevalence of IMHs in a Hispanic population. Its findings are similar to those published for other populations. The observed prevalence emphasizes the importance of proactive screening for IMHs in older patients, especially if they are women. Clinical markers such as the Watzke-Allen sign are helpful in the early detection of IMHs.

## Introduction

An idiopathic macular hole (IMH) is a foveal opening in the neurosensory retina caused by perifoveal vitreomacular traction and detachment [1]. Typical symptoms include metamorphopsia and central scotomas that vary as holes evolve. IMHs progress through clinically defined stages over weeks to months [2,3]. Advances in optical coherence tomography (OCT) have allowed for more precise imaging and classification of macular holes, improving diagnostic accuracy and treatment planning [4,5].

To our knowledge, no studies have examined the prevalence of IMHs in a Hispanic population. Investigating the prevalence, spectrum, sex ratio, and age of presentation of IMHs in this demographic provides insight into the disorder's disease burden and data for healthcare planning. Additionally, there is a dearth of evidence regarding the use of OCT testing for IMHs in Hispanic populations. Understanding racial and ethnic disparities in retinal conditions, including IMHs, has become increasingly important for addressing inequities in eye care delivery and outcomes [6,7]. The purpose of this study is to determine the prevalence of IMHs and related OCT testing in a Hispanic population.

## Materials and methods

Study design and setting

This retrospective, descriptive, cross-sectional study was conducted at the Institute of Ophthalmology Fundacion Conde de Valenciana, a large referral center, over a four-year period from March 2018 to July 2022. The study followed the Strengthening the Reporting of Observational Studies in Epidemiology (STROBE) guidelines [8].

Participants

**Figure 1:**
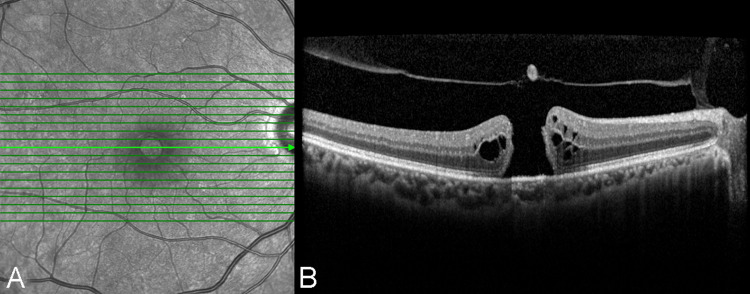
Idiopathic macular hole (A) Infrared image and (B) OCT B-scan demonstrating a typical idiopathic macular hole. The images show a complete opening in the neurosensory retina, with intraretinal cysts at the edges of the hole. Additionally, the retinal operculum remains attached to the vitreous. OCT: optical coherence tomography

Electronic records of patients aged 18 years and above were reviewed. All patients who presented for a first-time comprehensive ophthalmology evaluation were included. The diagnosis of IMH (Figure 1) was made clinically and was confirmed with OCT. Exclusion criteria included secondary macular hole, cataract (Lens Opacities Classification System Version III (LOCS III) classification > NC3), high myopia (< -6.0D and/or axial length > 26 mm), diabetic and hypertensive retinopathy, macular edema, glaucoma, lamellar hole or pseudo-hole, previous ocular trauma, macular telangiectasia, choroidal neovascularization, foveal hypoplasia or fovea plana, prior Irvine-Gass syndrome, retinal dystrophies, and any cause of decreased vision other than IMH.

Data collection

Variables analyzed included gender, arterial hypertension, type 2 diabetes mellitus, lens status (phakic/pseudophakic), metamorphopsia (evaluated using Amsler grid), visual acuity (Snellen chart), intraocular pressure (measured with a Goldmann tonometer), slit lamp examination findings of the anterior segment, ophthalmoscopy findings under pharmacologic dilation (tropicamide 50 mg and phenylephrine 8 mg, two drops in both eyes), and baseline macular OCT findings (Spectralis, Heidelberg Engineering, Heidelberg, Germany) of 31 B-scans in a 25 × 30 area, with each high-resolution horizontal B-scan separated by a distance of 240 μm.

Data analysis

Data from Snellen chart testing was used to assess visual acuity. Equivalent logarithm of the minimum angle of resolution (LogMAR) data were computed for statistical analysis. Prevalence was calculated using the following formula: (N subjects with idiopathic macular hole / M subjects who attended consultation for the first time (2018-2022) × 100%) or (397/116655×100%). Poisson confidence intervals of 95% by substitution methods were calculated for prevalence and ratios. Intraocular pressure was measured in millimeters of mercury (mmHg). Retinal layers were classified using the INᐧOCT consensus lexicon [9]. Descriptive statistics included frequencies and percentages for qualitative variables and measures of central tendency and dispersion for quantitative variables. Inferential statistics involved normality tests (Kolmogorov-Smirnov). Quantitative variables were reported as mean and standard deviations (SD). Subgroups, due to categorical variable analysis, were analyzed using Pearson's X2 and independent T-tests. Univariate analysis was done for odds ratios. The statistical significance used was p < 0.05. Data were analyzed using SPSS Statistics for Windows version 29.0 (IBM Corp., Armonk, NY).

Ethical considerations

The study adhered to ethical standards outlined in the Helsinki Declaration of 1975 and the 17th article of law regulations in general health. Protocol CI-063-2022 was registered for review by the board of the Autonomous University of Mexico (UNAM) and COFEPRIS under the code 17CI09015008. The Ethics Committee of the Institute of Ophthalmology Fundacion Conde de Valenciana issued approval IOFCV-Ret2024101.

## Results

A total of 116,655 patients had initial visits at the Institute of Ophthalmology Fundacion Conde de Valenciana between 2018 and 2022. Out of these, 1,368 patients were initially suspected to have IMH based on clinical appearance. However, OCT confirmed the presence of IMH in only 397 patients. The remaining patients were diagnosed with conditions such as epiretinal membrane, vitreoretinal traction, or lamellar macular holes. The clinical characteristics and comorbidities in patients with IMH are summarized in Table 1.

**Table 1:** Clinical characteristics and comorbidities in patients with idiopathic macular hole

Variable	Number (%)
Female	318 (80.1%)
Male	79 (19.9%)
Right eye	202 (50.9%)
Left eye	195 (49.1%)
Type 2 diabetes mellitus	86 (21.75%)
Arterial hypertension	169 (42.5%)
Metamorphopsias	94 (23.7%)
Central scotoma	23 (5.80%)
Watzke-Allen sign	218 (79.8%)
Pseudophakic	43 (10.8%)

The estimated prevalence of IMH was 0.34% (95% confidence interval (CI): 0.307-0.375). Females constituted a preponderance of the study population, comprising 318 (80%) of the 397 subjects, with a female-to-male ratio of 4:1. The mean age of the participants was 67.75 ± 9.38 years. There was no significant difference between genders.

Initial LogMAR visual acuity was 1.04 ± 0.58, with no significant differences for gender, symptoms, or clinical findings. IMH incidence was similar in right (202 subjects, 50.9%) and left eyes (195 subjects, 49.1%). Type 2 diabetes mellitus was present in 86 (21.75%) subjects. Arterial hypertension was present in 169 (42.5%) subjects. Notably, 86 (21.75%) subjects had both type 2 diabetes mellitus and arterial hypertension. Metamorphopsia was reported by 94 (23.7%) subjects and central scotomas by 23 (5.8%) subjects. The Watzke-Allen sign was present in 318 (79.8%) subjects. Forty-three (10.8%) eyes were pseudophakic.

There was a significant association between patients reporting metamorphopsia and those who were found to have a positive Watzke-Allen sign, with an odds ratio of 3.92 (95% CI: 2.32-6.63, p < 0.001). Similarly, patients with a positive Watzke-Allen sign had a central scotoma odds ratio of 2.23 (95% CI: 0.91-5.47, p = 0.071). Subgroup analyses revealed no significant gender differences for age, visual acuity, comorbidities (arterial hypertension and type 2 diabetes mellitus), or visual acuity. Age was significantly lower in patients without hypertension, pseudophakia, or central scotomas.

## Discussion

Previously reported IMH prevalence varies in different populations from 0.02% to 0.8% [10,11]. The Beaver Dam and Baltimore Eye Studies found prevalences of 0.29% and 0.33%, respectively [12,13]. The Australian Blue Mountains and Chinese Beijing Eye Studies found lower prevalences of 0.02% and 0.09%, respectively [10,14]. An IMH prevalence was reported to be 0.17% for a southern Indian population [15]. Table 2 summarizes this data. The rate of bilateral macular holes varied from 8% to 14.3% for Indian and Chinese populations, respectively [10,15]. Additionally, IMHs were more common in females, with female-to-male ratios ranging from 1.2:1 to 7:1 [10,15].

**Table 2:** Overview of prevalence studies on macular hole in various populations

Study	Number of study subjects	Objective	Methodology	Results (prevalence per 1,000)
The Baltimore Eye Study (1996) [9]	5,308	Estimate the cause-specific prevalence of visual impairment in an inner-city population	Descriptive, cross-sectional, population based-sample	0.33%
The Blue Mountain Eye Study (1997) [10]	3,490	Determine the prevalence and associations of epiretinal membranes in a defined older Australian population and assess their influence on visual acuity	Descriptive, population-based survey of vision and common eye diseases in a representative Australian urban population	0.02%
The Beijing Eye Study (2006) [6]	4,439	Determine the prevalence of full-thickness macular holes in the elderly Chinese population	Descriptive, cross-sectional population-based, cohort study	0.09%
Prevalence of idiopathic macular hole in adult rural and urban south Indian population (2008) [11]	7,774	Evaluate the prevalence of idiopathic macular hole in a southern Indian community	Descriptive, cross-sectional, population-based study with multistage sampling procedure	0.17%
The Beaver Dam Eye Study (2014) [8]	1,913	Describe the prevalence and interrelationships of epiretinal membranes, vitreomacular traction, macular cysts, paravascular cysts, lamellar macular holes, full-thickness macular holes, and visual impairment	Descriptive, longitudinal, observational, population-based study of older adults	0.29%

Our study's 0.34% IMH prevalence was similar to the Beaver Dam and Baltimore Eye Studies, which found prevalences of 0.29% and 0.33%, respectively [12,13]. We did find a higher female-to-male ratio of 4:1, perhaps due to later medical attention-seeking behavior among elderly men in our population. The delayed onset of symptoms associated with macular holes compared to other macular diseases underscores the importance of screening during ophthalmoscopic evaluation. Empirical assessments such as the Amsler grid and Watzke-Allen sign, both non-invasive and easily performed, were prevalent in our study cohort, highlighting their potential as clinical markers for prompting further diagnostic evaluation, such as macular OCT. The growing use of non-ophthalmic OCT instruments in geriatric studies of stroke and neurodegenerative diseases is a potential future screening resource.

In women presenting with sudden visual loss, metamorphopsia, and central scotoma, the presence of the Watzke-Allen sign was confirmed as a potential clinical indicator for initiating macular OCT evaluation. This highlights the importance of the clinical recognition of IMH's early clinical signs and symptoms to facilitate their timely diagnosis, prognostic assessment, and treatment planning.

A key strength of our study lies in its primary focus on the Hispanic population, in a specialized ophthalmological referral center that receives patients from all Mexican communities. Weaknesses include its single institution design, reduced patient recruitment during the pandemic era, and reliance on subjective patient responses for Amsler grid and Watzke-Allen tests. Future efforts should be directed toward refining diagnostic criteria and screening protocols for IMH, leveraging clinical markers such as the Watzke-Allen sign and community OCT resources to enhance detection rates and facilitate prompt intervention. Further research exploring the prognostic significance of early diagnostic indicators and the efficacy of treatment modalities will be valuable in optimizing IMH management strategies, improving patient outcomes, and allocating healthcare resources.

## Conclusions

Our study provides insight into the prevalence of IMHs in a Hispanic population. Its findings are similar to those published for other populations. The observed prevalence emphasizes the importance of proactive screening for IMHs in older patients, especially if they are women. Clinical markers such as the Watzke-Allen sign are helpful in the early detection of IMHs.
